# Effect of magnetic fields on cryptochrome-dependent responses in *Arabidopsis thaliana*

**DOI:** 10.1098/rsif.2008.0519

**Published:** 2009-02-25

**Authors:** Sue-Re Harris, Kevin B. Henbest, Kiminori Maeda, John R. Pannell, Christiane R. Timmel, P.J. Hore, Haruko Okamoto

**Affiliations:** 1 Department of Plant Sciences, University of Oxford, South Parks Road, Oxford OX1 3RB, UK; 2 Inorganic Chemistry Laboratory, Department of Chemistry, University of Oxford, South Parks Road, Oxford OX1 3QR, UK; 3 Physical and Theoretical Chemistry Laboratory, Department of Chemistry, University of Oxford, South Parks Road, Oxford OX1 3QZ, UK

**Keywords:** *Arabidopsis*, cryptochrome, extremely low-frequency electromagnetic fields, hypocotyl growth, magnetoreception, radical-pair mechanism

## Abstract

The scientific literature describing the effects of weak magnetic fields on living systems contains a plethora of contradictory reports, few successful independent replication studies and a dearth of plausible biophysical interaction mechanisms. Most such investigations have been unsystematic, devoid of testable theoretical predictions and, ultimately, unconvincing. A recent study, of magnetic responses in the model plant *Arabidopsis thaliana*, however, stands out; it has a clear hypothesis—that seedling growth is magnetically sensitive as a result of photoinduced radical-pair reactions in cryptochrome photoreceptors—tested by measuring several cryptochrome-dependent responses, all of which proved to be enhanced in a magnetic field of intensity 500 μT. The potential importance of this study in the debate on putative effects of extremely low-frequency electromagnetic fields on human health prompted us to subject it to the ‘gold standard’ of independent replication. With experimental conditions chosen to match those of the original study, we have measured hypocotyl lengths and anthocyanin accumulation for *Arabidopsis* seedlings grown in a 500 μT magnetic field, with simultaneous control experiments at 50 μT. Additionally, we have determined hypocotyl lengths of plants grown in 50 μT, 1 mT and approximately 100 mT magnetic fields (with zero-field controls), measured gene (*CHS*, *HY5* and *GST*) expression levels, investigated blue-light intensity effects and explored the influence of sucrose in the growth medium. In no case were consistent, statistically significant magnetic field responses detected.

## 1. Introduction

Exposure to the extremely low-frequency (ELF; 50/60 Hz) electromagnetic fields associated with electrical power distribution is unavoidable in modern industrial societies and has led to concerns about adverse effects on human health (Crumpton & Collins 2004; Crumpton 2005). On the basis of epidemiological evidence, which suggests a link between long-term exposure to ELF magnetic fields stronger than 0.4 μT and a small increased risk of childhood leukaemia (Ahlbom *et al*. 2000; Greenland *et al*. 2000), the International Agency for Research on Cancer has classified ELF magnetic fields as ‘possibly carcinogenic to humans’ (IARC 2002). The UK National Radiological Protection Board (now the Health Protection Agency), however, concluded in 2001 that there was no compelling evidence for carcinogenicity (AGNIR 2001). Despite much effort, laboratory-based studies have so far failed to establish convincing biological responses to weak ELF magnetic fields. Although plenty of apparently positive effects have been reported, the majority of studies have been unsystematic, there have been few serious attempts at independent replication, and most of those have failed to corroborate the original observations (Lacy-Hulbert *et al*. 1998; Galland & Pazur 2005; Pazur *et al*. 2007). Moreover, there are currently no plausible biophysical mechanisms that could explain how ELF fields weaker than 1 μT might significantly perturb a biological system or that could be used to guide experimental studies (Swanson & Kheifets 2006). Although the absence of an interaction mechanism cannot be taken to imply the absence of a response, it does increase the need for careful, well-designed and independently verified laboratory studies to establish whether there are ELF effects at the cellular or organism level.

Since its inception in the 1970s, the ‘radical-pair mechanism’ has become well established as the only known way in which magnetic interactions, many times smaller than the thermal energy per molecule (*k*_B_*T*), can alter the rates and yields of chemical reactions *in vitro* (Steiner & Ulrich 1989; Brocklehurst & McLauchlan 1996; Brocklehurst 2002; Woodward 2002; Timmel & Henbest 2004). The theoretical foundation of the mechanism is highly developed: experimental data can be interpreted quantitatively and magnetic field responses predicted from independently determined properties of the transient radical-pair intermediates (Rodgers *et al*. 2005, 2007). Most experimental studies have employed static magnetic fields, but with radical-pair lifetimes rarely in excess of a microsecond, one can expect essentially identical responses to slowly varying ELF fields (Scaiano *et al*. 1994). However, almost all investigations have employed magnetic fields stronger than approximately 1 mT and it has proved difficult to contrive experimental conditions in which much weaker magnetic fields have a significant chemical effect (Maeda *et al*. 2008).

Despite several reports of magnetic field effects on biomolecular radical pairs (Harkins & Grissom 1994; Taraban *et al*. 1997; Møller *et al*. 2000; Liu *et al*. 2005; Henbest *et al*. 2008), not all of which have met the test of independent replication (Jones *et al*. 2006, 2007), no such response has yet been convincingly established *in vivo*. However, it has been proposed that radical-pair reactions form the basis of the ability of migratory birds to sense the direction of the Earth's magnetic field for the purpose of orientation and navigation (Schulten *et al*. 1978; Ritz *et al*. 2000; Rodgers & Hore 2009). Supporting evidence has come from spectroscopic (Giovani *et al*. 2003; Liedvogel *et al*. 2007; Henbest *et al*. 2008) and theoretical (Weaver *et al*. 2000; Cintolesi *et al*. 2003; Solov'yov *et al*. 2007) studies and recent experiments on a model system (Maeda *et al*. 2008), which have shown for the first time that radical-pair reactions can respond measurably to magnetic field strengths weaker than 50 μT. Moreover, radiofrequency fields, which are known to modify radical-pair reaction yields *in vitro* (Woodward *et al*. 2001; Henbest *et al*. 2004; Rodgers *et al*. 2005), have been found to disrupt the magnetic compass of European robins at intensities below 1 μT (Ritz *et al*. 2004, in press; Thalau *et al*. 2005). These observations have been interpreted as diagnostic of a radical-pair magnetoreception mechanism.

The molecules proposed as the avian magnetoreceptors are cryptochromes—50–70 kDa blue-light photoreceptor flavoproteins—that regulate a variety of processes in organisms ranging from bacteria to humans (reviewed in Lin & Todo 2005; Partch & Sancar 2005; Losi 2007), and the only known source of potentially suitable radical pairs (reviewed in Mouritsen & Ritz 2005; Wiltschko & Wiltschko 2006; Johnsen & Lohmann 2008). Evidence in support of the cryptochrome hypothesis is gradually accumulating (Möller *et al*. 2004; Mouritsen *et al*. 2004), and includes a report of magnetic field responses in plants in which two cryptochromes, Cry1 and Cry2, mediate a number of photoresponses, including blue-light growth inhibition and entrainment of the circadian clock (Ahmad *et al*. 2007). Enhanced cryptochrome-mediated inhibition of hypocotyl (stem) elongation in *Arabidopsis thaliana* in a 500 μT magnetic field, relative to controls in the ambient magnetic field (approx. 40 μT), was observed under blue-light irradiation but not under red light (where the mediating photoreceptors are phytochromes), nor in total darkness (Ahmad *et al*. 2007). No magnetic field effects were found in *Cry1*/*Cry2*-deficient *Arabidopsis* mutants. Blue light-induced degradation of Cry2 and blue light-dependent anthocyanin accumulation (another cryptochrome-dependent process) were also enhanced at 500 μT. All three effects of the 500 μT field—a 12–37 per cent reduction in hypocotyl lengths, a 28–45 per cent increase in anthocyanin production and a reduction in Cry2 levels—are consistent with a magnetic field-induced increase in the sensitivity of the seedlings to blue light. This was interpreted by Ahmad *et al*. in terms of a flavin–tryptophan radical pair formed by photoinduced electron transfer within a cryptochrome photoreceptor (Giovani *et al*. 2003).

Although the *Arabidopsis* study was not motivated by health issues, it is significant on several counts which distinguish it from the vast majority of the literature on putative biological responses to weak magnetic fields. The responses were not large but they were observed in two laboratories (Paris and Frankfurt; Ahmad *et al*. 2007) using more than one experimental measure. Unusually for a study of biological magnetic responses, there was a proposed biophysical mechanism and a proposed receptor, allowing various theoretical predictions to be tested. Though the experiments were at field strengths much higher than human ELF exposures, *A. thaliana* appears to be a promising model for the investigation of biological responses to weak ELF and static fields. If plants, for which magnetic responses have no apparent function and which presumably lack a highly evolved, specialized magnetoreceptor, are sensitive to external magnetic fields then conceivably other cryptochrome-containing species may be too. In this context, it may be noted that cryptochromes are key components in the regulation of the mammalian circadian clock and that disruption of circadian timing has been linked to susceptibility to cancer (Reddy *et al*. 2005).

We report here our attempts to subject the *Arabidopsis* study to the ‘gold standard’ (Crumpton 2005) of independent replication. Choosing experimental conditions to match those used in the original study, we have compared hypocotyl lengths and anthocyanin accumulation of seedlings grown in a 500 μT magnetic field with controls at 50 μT. Additionally, hypocotyl lengths of plants grown in 0 μT, 1000 μT and approximately 100 mT magnetic fields have been recorded, gene (*CHS*, *HY5* and *GST*) expression levels measured, blue-light intensity effects investigated and the influence of sucrose in the growth medium explored.

## 2. Materials and methods

### 2.1. Plant materials and growth conditions

*Arabidopsis thaliana* Landsberg *erecta* wild-type seeds were surface sterilized with 70 per cent ethanol containing 10 per cent sodium hypochlorite (BDH, Poole, UK) and air-dried on filter paper (Whatman, UK). The seeds were individually sown on the surface of agar plates (5 cm diameter and 2 cm height; Sterilin, Caerphilly, UK) containing half-strength Murashige and Skoog medium including vitamins, buffered to pH 5.7 using MES-KOH (Melford, Chelsworth, The Netherlands) and 0.8 per cent agar (Sigma, St Louis, MO, USA). All experiments were performed with 2 per cent sucrose (BDH) in the growth media, except for those in which plants were grown under 3 W m^−2^ illumination, for which no sucrose was present. The plates were sealed with Micropore tape (3M Health Care, Munich, Germany) to allow gas exchange and to avoid condensation, and were kept in a light-tight container at 4°C for either 48 hours (in almost all the experiments) or else 24 hours. The seeds were then incubated for 24 hours at 21°C (±1.5°C) under homogeneous and continuous irradiation from a fluorescent light source (L36W/21-840 Coolwhite; Osram, Germany) at approximately 16 W m^−2^ before being transferred to darkness at 21°C (±1.5°C) until the radicles (the embryonic roots) had emerged (typically 40 hours with 2% sucrose; 20 hours without). The plates were then exposed to continuous blue-light irradiation for 72 hours at 21°C (±0.5°C) in a temperature-controlled plant growth chamber, either a Microclima 1000 (Snijders Scientific, Tilburg, The Netherlands) or an E-30 light-emitting diode (LED; Percival Scientific, Boone, IA, USA). This contrasts with the study of Ahmad *et al*. (2007) in which the exposures were performed on a bench in an air-conditioned room. Other minor differences in procedure are that in the original study, a temperature of 5°C was used for the initial 48 hours dark period and the plates were irradiated for ‘typically 24 hours’ during a 48 hours dark period at room temperature to induce germination (Ahmad *et al*. 2007).

In our study, the seeds were harvested from plants grown individually on peat plugs (Jiffy-7; Jiffy International Products, Kristansand, Norway) in a greenhouse in Oxford at 21°C (approx. ±4°C), with supplemental lighting. All the seeds used in this study were harvested within the space of a year and showed synchronized 100 per cent germination in every experiment. Except where indicated, replicates of each measurement were performed sequentially under identical conditions using seeds harvested from the same plant. When comparing the hypocotyl lengths of seedlings grown in 50 and 500 μT magnetic fields, measurements were repeated using seeds harvested from several individual plants to ensure that the results were not specific to parental growth conditions.

The seedlings used for RNA preparation were grown as described above. The gene expression data are the means of three biological replicates comprising 10 seedlings each, harvested from the same plate.

### 2.2. Light sources

For the measurements in which the magnetic field was generated with Helmholtz coils (§2.3), monochromatic blue light (470±10 nm) was provided by arrays of LEDs (LXHL-MBJA 18 LED Flood; Luxeon, CA, USA). A homogeneous light intensity of 20 W m^−2^ across the surface of the 5 cm plate was obtained using diffusers placed between the light source and the plate (see below for details). Initial experiments used arrays of blue LEDs (465±10 nm; Conrad) identical to those of Ahmad *et al*. (2007), but were replaced at an early stage by the Luxeon LED arrays which delivered more homogeneous illumination and had better long-term output stability. To achieve a light intensity of 20 W m^−2^ from the Conrad LED arrays, we required currents in excess of the maximum recommended for these diodes. This was probably the origin of the reduced light output measured at the end of a 72 hours experiment compared with that at the beginning. Given that the flavin absorption band of cryptochrome, centred at approximately 450 nm, has a width of approximately 100 nm, it seems very unlikely that the different, but strongly overlapping, wavelength bands of the two types of diode could make a significant difference.

For the experiments performed with permanent magnets, monochromatic blue light (470±10 nm) was provided by the LEDs supplied with the Percival E-30 LED chamber. Homogeneous light intensities (12 and 3 W m^−2^; ±10%) were obtained by varying the distance of the plates from the light source and by interposing a layer of filter paper (Whatman, UK).

Light intensities were measured with SKP 200 photometers (Skye-Instruments, Powys, UK) before and after every 72 hours exposure. No differences were found for the Luxeon LED arrays.

### 2.3. Magnetic fields

Two identical pairs of Helmholtz coils (for simultaneous exposure and sham measurements) were wound on formers manufactured from the hard, non-magnetic plastic Delrin. Each Helmholtz pair consisted of two doubly wound coils of copper wire, 2×150 turns, 44 cm diameter and 22 cm separation. These dimensions ensured a uniform magnetic field (to within 98%) throughout the two Petri dishes placed in the centre of the coils. The intensities of the static magnetic fields were set at fixed values between 0 and 1000 μT (to a precision of ±10 μT) by supplying the appropriate direct current to each coil (typically approx. 1 A). The exposure and sham conditions were generated by running identical currents in parallel or antiparallel, respectively, through the doubly wound coils, to ensure that any small heating effects were the same for the two sets of coils. The LEDs irradiated the plates from a fixed height of 9 cm above the sample stage. The light passed through three diffusers: two thin plastic discs placed 1 cm below the LED array and, 2 cm below them, a white Perspex plate of thickness 2 mm, allowing homogeneous irradiation (95%) across the 5 cm diameter plates. The currents supplied to the LEDs were calibrated to ensure identical irradiation intensities in the two sets of coils. The magnetic fields had no measurable effect on the light output of the LEDs. Magnetic fields were measured using fluxgate magnetometers (Macintyre Electronic Design Associates, Inc., Dulles, VA, USA) and a gaussmeter (F. W. Bell, model 4048003 fitted with a 1778 Hall probe sensor) before and after every experiment. Magnetic field intensities close to that of the Earth were measured with a μMAG series handheld fluxgate magnetometer (Meda Inc., Dulles, VA, USA) sensitive to ±1 nT. The two sets of coils (with an approx. 60 cm centre-to-centre horizontal separation) were magnetically shielded from one another by a vertical 1 mm sheet of mu-metal (Magnetic Shield Ltd, Surrey, UK). No field (to within 10 μT) was detectable at the centre of one coil as a result of its neighbour. The time-dependent magnetic fields in the frequency range of up to 100 Hz at the positions of the samples, e.g. from the compressor in the base of the Microclima chamber, were measured and found to be weaker than 5 nT. Two plates were stacked in the centre of each pair of coils; the upper was irradiated with blue light while the lower, wrapped in aluminium foil, served as a dark control. The aluminium foil produced no attenuation of the applied magnetic field.

The magnetic field inside the Microclima chamber, with no current flowing in the coils, was found to be 0±10 μT, implying efficient shielding from the Earth's magnetic field (approx. 50 μT), and allowing control experiments to be performed in zero field. For these experiments, the currents supplied to the two Helmholtz pairs were either parallel, denoted as (+*A*, +*A*), or antiparallel, denoted as (+*A*, −*A*), the former being the exposure condition and the latter the zero-field control. To obtain a 500 μT (exposure)/50 μT (control) condition, (+*A*, +*A*) was used for the former and (+*A*+*α*, −*A*), with *α*≪*A*, for the latter. This ensured that the two coil sets carried very similar currents so that any temperature differences were kept to a minimum. Temperatures were measured over a 72 hours period at the position of the plates. The two sets of coils were found to have identical and stable temperatures (21±0.5°C) when the currents were flowing. In the experiments of Ahmad *et al*. (2007), the coils were tilted to ensure that the applied field was collinear with the local geomagnetic field. Given the efficient magnetic shielding afforded by our Microclima chamber, this alignment was not felt to be necessary. However, all experiments reported here were performed with the coil axes tilted (by 24° from the vertical) in case the effects of the applied field depended on its direction with respect to gravity. Ahmad *et al*. (2007) do not specify the tilt angle or whether different angles were used in Paris and Frankfurt. We chose 24° to match the geomagnetic inclination in Frankfurt (Ritz *et al*. 2004; Thalau *et al*. 2005), where the coils appear to have been constructed.

Permanent neodymium magnets (2.5 cm diameter, 1 cm height, First4magnets, Birmingham, UK) were used for experiments at higher magnetic field strengths. The two plates were stacked as described above, with the magnet sandwiched between them. The mean magnetic field at the position of the seedlings was approximately 100 mT. For the control experiments, the magnet was replaced by a non-magnetic austenitic stainless steel 304L disc of identical dimensions.

In contrast to the study of Ahmad *et al*. (2007), all plant growth and reverse transcriptase polymerase chain reaction (RT-PCR; §2.4) measurements were performed with the experimenter blind to the magnetic exposure/sham control conditions (which were independently and randomly chosen for each experiment) until after the analysis had been completed. In some experiments, the two sets of coils were interchanged to check for any unexpected asymmetry.

### 2.4. RNA preparation and semi-quantitative RT-PCR

RNA was prepared using QIAshredder and RNeasy miniprep columns (Qiagen, Hilden, Germany) according to the manufacturer's protocol. cDNA was synthesized using the TaqMan reverse transcriptase system (Applied Biosystems Inc., Foster City, CA, USA). Each RT reaction contained cDNA, 2 pmol of each primer and 1×PowerSYBR Green (Applied Biosystems Inc.) in a final volume of 10 μl and the RT-PCR was performed in an ABI PRISM 7300 Sequence Detection System (Applied Biosystems Inc.). Primers used were as follows. *Tubulin5* (At1g20010; forward (5′-TGAATGCATGGTCCTCGACA-3′) and reverse (5′-GCAAGTCACACCGCTCATTGT-3′)); *CHS* (At5g13930; forward (5′-GGCTCAGAGAGCTGATGGAC-3′) and reverse (5′-CATGTGACGTTTCCGAATTG-3′)); *HY5* (At5g11260; forward (5′-ATCAAGCAGCGAGAGGTCAT-3′) and reverse (5′-CGACAGCTTCTCCTCCAAAC-3′)); and *GST* (At1g10370; forward (5′-AACCGGTGAGTGAGTCCAAC-3′) and reverse (5′-AGCGACAAACCACTTTTCGT-3′)). Primers were designed over an intron to avoid detecting the genomic DNA fragments. Baseline data were collected between cycles 3 and 15 and an *R*_*n*_ threshold of 0.35 was used for all amplification plots to obtain *C*_T_ (threshold cycle) values. Standard curves were obtained by the dilution of every cDNA sample. In order to compare the data from different PCR runs or cDNA, *C*_T_ values were normalized to the *C*_T_ values of *tubulin5* and were used to calculate the relative gene expression per 1 ng RNA.

### 2.5. Anthocyanin accumulation

Anthocyanin accumulation was measured using seedlings grown under 12 W m^−2^ blue light for 48 hours at 21±0.5°C in the presence of 50 or 500 μT magnetic fields. Anthocyanin was extracted by grinding 20 seedlings in 300 μl 1 per cent HCl in methanol and incubating at room temperature in darkness for more than 6 hours. Twice-distilled H_2_O (200 μl) was added to the extract and chlorophyll was extracted using an equal volume of chloroform. The relative amount of anthocyanin in the aqueous phase was estimated using measured values of the optical absorption at 530 and 657 nm according to *A*_530_−0.25*A*_657_ (Rabino & Mancinelli 1986). Each experiment comprised at least three measurements using 20 seedlings each time. Minor differences in the procedure from Ahmad *et al*. (2007) are: a smaller solvent volume was used to obtain a more concentrated sample, and thus a more precise determination of the anthocyanin concentrations; a longer incubation time was used to ensure that all the pigment was extracted; fixed (48 hours instead of 48–55 hours) seedling growth times and fixed (6 hours instead of 3–4 hours) dark incubation times were used. It is very unlikely that any of these small differences would significantly have affected the results. Nor does it seem probable that the difference in the temperature at which the seedlings were grown (21°C instead of 25–26°C) is important. The anthocyanin accumulation in blue light is 0.13±0.01 at 21°C (arbitrary units) and 0.11±0.02 at 25°C. It therefore seems unlikely that the different growth temperatures would result in very different magnetic field effects.

### 2.6. Statistical analysis

Immediately after the 72 hours magnetic field exposure, all the seedlings on the experimental plates were laid flat on the surface of a new agar plate. Hypocotyl lengths were measured (±0.02 mm) from a scanned image of the seedlings using ImageJ (http://rsb.info.nih.gov/ij/) software. The mean hypocotyl length for each dish was used in a two-tailed paired *t*-test analysis to compare the growth of seedlings exposed to the magnetic field with those grown simultaneously under the control conditions, using a 95% confidence level (*p*<0.05) to judge statistical significance.

## 3. Results

### 3.1. Hypocotyl growth in 50 and 500 μT fields

**Figure 1:**
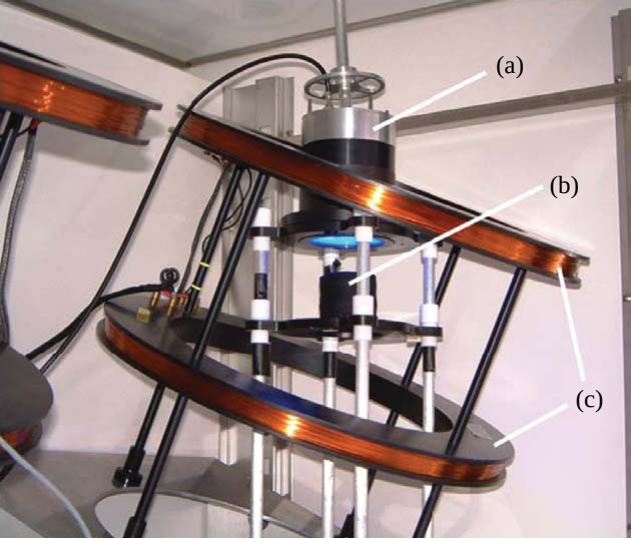
Apparatus used for the exposure of *A. thaliana* seedlings to 0–1000 μT magnetic fields, showing (a) the assembly that houses the light-emitting diode array and diffusers, (b) the plates of seedlings, and (c) the Helmholtz coils tilted at 24° to the vertical, inside the plant growth chamber. Parts of the other set of coils are visible on the left. The mu-metal screen between the two sets of coils has been removed for this photograph.

Experiments in which wild-type *A. thaliana* seedlings were grown in 50 μT (‘control’) or 500 μT (‘exposure’) static magnetic fields were performed at 21°C using two identical sets of doubly wound Helmholtz coils, placed side by side in a temperature-controlled plant growth chamber (figure 1). The effect of the magnetic field on seedlings grown in darkness was tested using a Petri dish covered with aluminium foil placed immediately beneath the dish exposed to the blue light. Except where otherwise stated (see also §2), experimental conditions were as close as possible to those of Ahmad *et al*. (2007).

Three sets of experiments, denoted *A*, *B* and *C*, were performed, comprising 3, 3 and 5 replicates, respectively, each using seeds from a different plant, grown and harvested at different times of the year. The results are shown in table 1. In contrast to the study of Ahmad *et al*. (2007), the mean hypocotyl lengths at 500 μT in these 11 measurements were not consistently shorter than those at 50 μT. The differences in the mean lengths in the two fields (control minus exposure) varied from +8 to −10 per cent under blue-light conditions and were considerably smaller than the approximately +30 per cent change found previously (Ahmad *et al*. 2007). Two-tailed paired *t*-tests were performed on the 11 measurements taken together, using the mean hypocotyl length per dish as the measure (table 2). There was no statistically significant effect of the field on the dark-grown seedlings (*p*=0.535), in agreement with Ahmad *et al*. (2007). The differences in seedling growth in the two fields under blue-light conditions were also not significant (*p*=0.396). When the three sets of data were analysed separately (table 2), set *B* alone was found to have a *p*<0.05 (*p*=0.013); for these experiments, the mean hypocotyl length was 5 per cent *longer* at 500 μT than at 50 μT, i.e. in the opposite direction to that found by Ahmad *et al*. (2007). When these four analyses were repeated using a one-tailed paired *t*-test to judge whether the mean hypocotyl lengths are significantly *shorter* (instead of significantly different) at 500 μT, only set *C* had *p*<0.05 (*p*=0.043).

**Table 1:** Hypocotyl lengths in 50 and 500 μT magnetic fields. (Seedlings were germinated and were grown under either blue light (20 W m^−2^) or in darkness for 72 hours. s.d., standard deviation; no. of seedlings is the number in each dish; *Δ*=100×(mean control length−mean exposure length)/mean control length.)

	blue light					darkness				
	control (50 μT)		exposure (500 μT)			control (50 μT)		exposure (500 μT)		
	mean length (±s.d.)/mm	no. of seedlings	mean length (±s.d.)/mm	no. of seedlings	*Δ*/%	mean length (±s.d.)/mm	no. of seedlings	mean length (±s.d.)/mm	no. of seedlings	*Δ*/%
*A1*	4.71 (±0.79)	45	4.34 (±0.72)	45	+8	7.92 (±1.19)	17	8.54 (±1.27)	27	−8
*A2*	3.83 (±0.66)	40	4.20 (±0.89)	36	−10	9.63 (±1.43)	24	8.77 (±1.53)	24	+9
*A3*	4.41 (±0.52)	29	4.23 (±0.69)	40	+4	9.31 (±1.27)	27	9.60 (±1.90)	27	−3
*B1*	2.72 (±0.52)	31	2.87 (±0.41)	41	−6	7.26 (±0.88)	35	7.62 (±0.74)	35	−5
*B2*	2.57 (±0.38)	32	2.67 (±0.40)	33	−4	7.46 (±0.76)	30	8.01 (±0.96)	26	−7
*B3*	2.08 (±0.34)	20	2.21 (±0.32)	19	−6	6.85 (±1.14)	17	7.05 (±1.34)	18	−3
*C1*	5.28 (±0.75)	33	4.94 (±0.83)	24	+6	9.96 (±1.44)	26	9.97 (±1.54)	28	0
*C2*	4.98 (±0.65)	33	4.90 (±0.77)	27	+2	10.12 (±1.53)	24	10.19 (±1.61)	18	−1
*C3*	5.79 (±0.77)	34	5.55 (±0.81)	34	+4	10.34 (±2.17)	21	11.58 (±1.78)	25	−12
*C4*	5.47 (±0.95)	37	5.55 (±0.92)	38	−1	11.00 (±2.65)	38	10.10 (±2.97)	35	+8
*C5*	6.15 (±1.46)	37	5.80 (±1.09)	35	+6	9.54 (±1.75)	30	9.48 (±2.78)	34	+1

**Table 2:** Paired *t*-test analysis of hypocotyl lengths in 50 and 500 μT magnetic fields. (The Paris and Frankfurt data were taken from Ahmad *et al*. (2007). *n*, number of replicates; *Δ*′=(mean control length−mean exposure length); *ABC*, analysis of the combined data from experiments *A*, *B* and *C*. *p<0.05.)

				blue light					darkness				
location	magnetic field control/exposure	experiment	light intensity/W m^−2^	*n*	mean (50 μT)/mm	mean (500 μT)/mm	*Δ*′/mm	*p*-value	*n*	mean (50 μT)/mm	mean (500 μT)/mm	*Δ*′/mm	*p*-value
Oxford	50/500 μT	*ABC*	20	11	4.36	4.30	0.07	0.396	11	9.04	9.17	−0.14	0.476
		*A*	20	3	4.32	4.26	0.06	0.812	3	8.95	8.97	−0.02	0.974
		*B*	20	3	2.46	2.58	−0.13	0.013*	3	7.19	7.56	−0.37	0.067
		*C*	20	5	5.53	5.35	0.19	0.087	5	10.19	10.26	−0.07	0.843
Paris	∼50/500 μT		20	3	3.39	2.38	1.01	0.005*^a^	3	9.69	9.79	−0.10	0.362
			12	3	4.61	3.12	1.49	0.007*^a^					
Frankfurt	∼50/500 μT		20	3	4.05	3.24	0.81	0.107	3	7.98	7.65	0.33	0.195
			3.3	3	6.80	5.61	1.19	0.221					

^a^Significance after Bonferroni correction (Rice 1989).

### 3.2. Anthocyanin accumulation in 50 and 500 μT fields

The accumulation of the flavonoid compound anthocyanin is cryptochrome dependent under blue-light irradiation (Briggs & Olney 2001; Ahmad 2003) and was found to be enhanced by up to 40 per cent in the presence of a 500 μT magnetic field (Ahmad *et al*. 2007). Five independent experiments were performed comparing anthocyanin levels in seedlings exposed to 500 μT fields, with 50 μT as the control (table 3). A two-tailed paired *t*-test revealed no significant difference between the two magnetic field conditions (*p*=0.531).

**Table 3:** Anthocyanin accumulation in 50 and 500 μT magnetic fields. (*Δ*=100×(mean control value−mean exposure value)/mean control value. A two-tailed paired *t*-test analysis of these data gave *p*=0.531.)

replicate	control (50 μT)	exposure (500 μT)	*Δ*/%
1	0.212	0.210	+1
2	0.134	0.126	+6
3	0.195	0.215	−10
4	0.164	0.139	+15
5	0.115	0.105	+9

### 3.3. Gene expression in 50 and 500 μT fields

Measurements of cryptochrome-regulated expression of chalcone synthase (*CHS*), which encodes an enzyme for anthocyanin biosynthesis, and of *HY5* were made in an attempt to find a more sensitive response to the applied magnetic field. The *HY5* gene encodes a bZIP transcription factor that binds to the G-box element in the promoter (Chattopadhyay *et al*. 1998) and regulates a group of blue light and cryptochrome-regulated genes including *CHS* (Ma *et al*. 2001; Ohgishi *et al*. 2004; Kleine *et al*. 2007). Accordingly, semi-quantitative RT-PCR was performed on seedlings grown for 72 hours under blue light in a 500 μT field using 50 μT as the control. The results (table 4) indicate that the mean expression levels of both *CHS* (*p*=0.863) and *HY5* (*p*=0.639) are indistinguishable for seedlings grown under the two magnetic field conditions.

**Table 4:** Gene expression levels. (*Δ*=100×(mean control value−mean exposure value)/mean control value. *p<0.05.)

magnetic field control/exposure	light intensity/W m^−2^	sucrose conc./%	gene	control expression level	exposure expression level	*Δ*/%	*p*-value
50/500 μT	20	2	*CHS*	1.274	0.977	+2	0.863
				1.193	1.335		
				0.996	1.071		
			*HY5*	0.345	0.265	+2	0.639
				1.563	1.585		
				0.229	0.235		
0/100 mT	20	2	*CHS*	2.710	4.663	−47	0.390
				1.628	0.736		
				2.433	4.585		
			*HY5*	0.094	0.097	−3	0.572
				0.093	0.101		
				0.092	0.088		
			*GST*	0.522	0.599	−19	0.021*
				0.476	0.602		
				0.534	0.627		
0/100 mT	12	2	*CHS*	2.110	2.551	−11	0.560
				1.524	1.482		
			*HY5*	0.140	0.145	−1	0.844
				0.085	0.082		
			*GST*	0.560	0.659	4	0.894
				0.476	0.337		
0/100 mT	3	0	*CHS*	0.329	0.203	12	0.294
				0.396	0.353		
				0.619	0.625		
			*HY5*	0.090	0.142	−16	0.282
				0.198	0.212		
				0.144	0.145		
			*GST*	0.476	1.008	−40	0.465
				0.451	0.410		
				0.299	0.302		

### 3.4. Hypocotyl growth in 0, 50 and 1000 μT fields

Radical-pair reactions investigated *in vitro* at different intensities of the applied magnetic field show a dose-response curve that is often not monotonic or even monophasic (Timmel *et al*. 1998). If the conditions of our experiments were subtly different from those employed in Paris and Frankfurt (Ahmad *et al*. 2007), it is conceivable that the Oxford seedlings had, by chance, essentially identical non-zero responses to 500 and 50 μT applied fields. There are also situations in which very long-lived radical pairs can respond rather differently at 50 and 0 μT (Timmel *et al*. 1998). To explore the field dependence of hypocotyl growth in a little more detail, we compared, separately, 50 and 1000 μT exposures with 0 μT controls (table 5). As shown in table 6, no significant differences were found, either in blue light or darkness, using a two-tailed paired *t*-test.

**Table 5:** Hypocotyl lengths in 50 μT, 1000 μT and 100 mT magnetic fields. (Seedlings were germinated and were grown under either blue light (20, 12 or 3 W m^−2^) or in darkness for 72 hours. s.d., standard deviation; no. of seedlings is the number in each dish; *Δ*=100×(mean control length−mean exposure length)/mean control length.)

			blue light					darkness				
			control		exposure			control		exposure		
magnetic field control/exposure	light intensity/W m^−2^	sucrose conc./%	mean length (±s.d.)/mm	no. of seedlings	mean length (±s.d.)/mm	no. of seedlings	*Δ*/%	mean length (±s.d.)/mm	no. of seedlings	mean length (±s.d.)/mm	no. of seedlings	*Δ*/%
0/50 μT	20	2	3.30 (±0.41)	30	3.39 (±0.47)	30	−3	8.14 (±1.00)	18	7.63 (±0.69)	24	+6
			3.38 (±0.52)	29	3.03 (±0.53)	28	+10	8.66 (±1.40)	14	8.68 (±0.94)	27	0
			3.15 (±0.53)	42	2.98 (±0.54)	52	+5	8.41 (±1.18)	34	8.59 (±0.95)	52	−2
0/1000 μT	20	2	3.11 (±0.37)	49	3.31 (±0.49)	38	−6	8.62 (±1.09)	41	8.96 (±1.57)	34	−4
			3.42 (±0.50)	64	3.08 (±0.38)	44	+10	9.13 (±1.17)	37	9.01 (±1.02)	44	+1
			4.52 (±0.91)	56	4.42 (±0.91)	53	+2	9.17 (±1.38)	48	9.05 (±1.46)	44	+1
0/100 mT	20	2	2.88 (±0.59)	30	3.30 (±0.71)	30	−15	11.31 (±1.28)	29	9.69 (±1.42)	28	+14
			3.43 (±0.60)	29	4.42 (±0.67)	29	−29	11.41 (±1.37)	29	11.33 (±1.97)	28	+1
			3.73 (±0.66)	29	3.62 (±0.73)	29	+3	13.85 (±1.98)	20	11.81 (±1.64)	20	+15
0/100 mT	12	2	4.12 (±0.81)	27	3.67 (±0.66)	26	+11	13.73 (±1.65)	29	12.67 (±1.77)	30	+8
			3.90 (±0.82)	28	4.47 (±0.67)	29	−15	14.45 (±1.47)	28	14.76 (±2.00)	27	−2
			3.46 (±0.51)	28	3.32 (±0.73)	29	+4	12.91 (±1.49)	28	13.49 (±1.80)	28	−4
0/100 mT	3	0	3.42 (±0.69)	29	3.13 (±0.49)	28	+8	12.84 (±0.86)	29	12.49 (±0.94)	29	+3
			4.02 (±0.42)	27	3.52 (±0.44)	28	+12	12.00 (±1.21)	29	12.48 (±1.16)	29	−4
			4.33 (±0.66)	28	4.00 (±0.42)	30	+8	13.66 (±1.32)	24	14.27 (±1.03)	23	−4

**Table 6:** Paired *t*-test analysis of hypocotyl lengths in 50 μT, 1000 μT and 100 mT fields. (*n*, number of replicates; *Δ*′=mean control length−mean exposure length. *p<0.05.)

				blue light				darkness			
magnetic field control/exposure	light intensity/W m^−2^	sucrose conc./%	*n*	mean (control)/mm	mean (exposure)/mm	*Δ*′/mm	*p*-value	mean (control)/mm	mean (exposure)/mm	*Δ*′/mm	*p*-value
0/50 μT	20	2	3	3.277	3.133	0.14	0.378	8.403	8.300	0.10	0.669
0/1000 μT	20	2	3	3.683	3.603	0.08	0.659	8.973	9.007	−0.03	0.848
0/100 mT	20	2	3	3.347	3.780	−0.43	0.306	12.190	10.943	1.25	0.171
0/100 mT	12	2	3	3.827	3.820	0.01	0.984	13.697	13.640	0.06	0.921
0/100 mT	3	0	3	3.923	3.550	0.37	0.028*	12.833	13.080	−0.25	0.498

### 3.5. 100 mT magnetic field exposure

When searching for the effects of weak magnetic fields on the product yields of radical-pair reactions *in vitro*, it is often wise to perform the initial experiments in magnetic fields much stronger than the intrinsic magnetic interactions in the radicals (typically 1–10 mT), on the basis that the effects are often larger than in weaker fields. Hypocotyl growth studies were therefore performed using a small permanent magnet to provide a field of approximately 100 mT. The mean hypocotyl lengths of three replicates grown under 20 W m^−2^ blue light or in darkness (table 5) were not significantly different from those grown in zero field (table 6). Wild-type and *Cry1* and *Cry2* double-mutant seedlings, grown in 0 and 100 mT fields, were visually indistinguishable from one other (see figure 1 in the electronic supplementary material).

The expression of the genes *CHS*, *HY5* and *GST*, a glutathione S-transferase, was examined in the presence and the absence of a 100 mT field. Similar to *CHS* and *HY5*, *GST* is upregulated at high blue-light intensity, a process that is mediated by Cry1 (Kleine *et al*. 2007). Only *GST*, for which the mean expression level was 19 per cent lower at 100 mT than in the zero field, had a *p*<0.05 (*p*=0.021).

### 3.6. Light-intensity dependence

The effects of 500 μT magnetic fields on hypocotyl growth were investigated in the study of Ahmad *et al*. (2007) using blue-light intensities of 3.3, 12 and 20 W m^−2^. The results of the experiments performed in Paris (12 and 20 W m^−2^) showed a smaller scatter than those in Frankfurt (3.3 and 12 W m^−2^), but essentially similar effects were found at all three intensities (approx. 30% reduction in mean hypocotyl length at 500 μT compared with approx. 50 μT). Three replicates were performed at 12 W m^−2^ to determine whether there was a dependence on the light intensity under the conditions used in Oxford (table 5). No significant difference between 100 and 0 mT was found (table 6).

No significant effect of the magnetic field was found on the expression of *CHS*, *HY5* or *GST* (table 4).

### 3.7. Sucrose dependence

The presence of sucrose in the growth medium influences the inhibition of hypocotyl growth in *Arabidopsis* under far-red light conditions and induces anthocyanin accumulation and *CHS* expression (Whitelam *et al*. 1993; Dijkwel *et al*. 1997). To test for similar effects on cryptochrome-dependent hypocotyl growth inhibition, seedlings were grown without sucrose, with and without a 100 mT magnetic field, under 3 W m^−2^ blue light. Low light intensities were used to avoid saturation of the blue-light response, which is more sensitive in the absence of sucrose. With three replicates, the difference in mean hypocotyl lengths gave *p*=0.028 in a two-tailed paired *t*-test (tables 5 and 6). No significant effect of the magnetic field was found on the expression of *CHS*, *HY5* and *GST* (table 4).

## 4. Discussion

Ahmad *et al*. (2007) reported consistent enhancements of *A. thaliana* blue-light responses in a 500 μT field relative to controls in the ambient magnetic field, including approximately 30 per cent reduction in hypocotyl lengths and up to 45 per cent increase in anthocyanin production. The six blue-light growth experiments performed in Frankfurt (three each at 20 and 3.3 W m^−2^) and the six in Paris (three each at 20 and 12 W m^−2^) all showed shorter hypocotyls at the higher field. Although the absolute differences between the mean hypocotyl lengths were reasonably clear, the statistical levels of significance reported were based on an inappropriate number of degrees of freedom. This is because individual seedlings were used as replicates although they were grown within the same Petri dish and were therefore not independent of one another. Treating them as such violates a key assumption of the statistical tests used and leads to inflated type 1 error (Hurlbert 1984).

In the light of this uncertainty, we reanalysed the Paris and Frankfurt data using a paired *t*-test, treating Petri dish means as independent data points (table 2). Both of the Paris measurements (20 and 12 W m^−2^) gave a *p*<0.05 (0.005 and 0.007, respectively) for a two-tailed test. However, neither of the experiments performed in Frankfurt (20 and 3.3 W m^−2^) showed statistically significant differences (*p*=0.107 and 0.221, respectively, in a two-tailed paired *t*-test; *p*=0.053 and 0.110, respectively, in a one-tailed paired *t*-test). Ahmad *et al*. (2007) noted that, compared with the Paris experiments, the seedlings grown in Frankfurt show greater variation in the state of germination from one replicate to the next and thus a larger spread of responses to the magnetic field.

Our attempts to replicate the Paris/Frankfurt hypocotyl growth measurements (tables 1 and 2) and our additional experiments using different magnetic field intensities (tables 5 and 6) gave two apparently significant results: (i) 500 μT exposure, 50 μT control, 20 W m^−2^ blue light, 2 per cent sucrose, *p*=0.013 and (ii) 100 mT exposure, 0 mT control, 3 W m^−2^ blue light, no sucrose, *p*=0.028. In the former case, the hypocotyls were on average longer at the higher field; in the latter case, they were shorter. However, given the number of *t*-tests performed here, a few of the *p* values are likely, by chance, to be less than 0.05 even if the magnetic field has no effect on the growth of the seedlings, i.e. repeated tests inflate type 1 error. Nine sets of hypocotyl data obtained with blue-light irradiation have been analysed here (tables 2 and 5, excluding the Paris/Frankfurt data). Applying the Bonferroni correction (Rice 1989), the 95% confidence limit should be set at *p*=0.05/9≈0.0056, causing the apparent significance of the two above-mentioned experiments to fall away. It is, therefore, difficult to conclude that a magnetic field response is reliably detectable under the conditions employed in our experiments. A similar picture emerges from the other blue-light responses reported here. The magnetic field effect on anthocyanin accumulation was not significant, and only 1 out of 11 gene expression analyses gave *p*<0.05 (*GST*, 100 mT exposure, 0 mT control, 20 W m^−2^ blue light, 2% sucrose, *p*=0.021).

Except where otherwise stated, the experimental conditions in Oxford were chosen to match as closely as possible those employed in Paris and Frankfurt and were, we believe, at least as well controlled. The only major differences in the procedures were that (i) we performed the magnetic field exposures in a commercial temperature-controlled plant growth chamber (21±0.5°C) rather than in air-conditioned rooms whose temperatures varied between 20.5 and 22.7°C (Frankfurt), and between 19 and 21°C (Paris; Ahmad *et al*. 2007) and (ii) the set of coils used to generate the exposure and the control conditions was chosen randomly in each experiment, with the experimenter blind in every case to the arrangement until the analysis was complete. As well as avoiding unconscious bias on the part of the experimenter, this protocol minimizes systematic errors arising from correlations between the intensity of the blue light and the magnetic field strength generated by a particular combination of LEDs and coils. It seems unlikely that there could have been significant temperature differences between the exposed and control seedlings in both Paris and Frankfurt. The use of doubly wound coils should have ensured that any heating effects were the same for the two sets of coils.

A minor difference in conditions in the two studies is the magnetic field strength used for the control experiments: 50±10 μT in Oxford, compared with 44 μT in Frankfurt and 33 μT in Paris. Although, under extreme conditions, a radical-pair reaction could respond significantly differently to such similar magnetic field strengths (Timmel *et al*. 1998), it is unlikely that this could explain our failure to replicate the Paris/Frankfurt observations. Even if, by chance, the plants grown in Oxford had responded identically to 50 and 500 μT fields, it seems improbable that all the other exposure conditions employed here (0/50 μT, 0/1000 μT or 0/100 mT) suffered a similar coincidence.

A possible reason for the absence of a response at a particular magnetic field intensity would be the fortuitous cancellation of the effects of the Δ*g* and hyperfine mechanisms of singlet–triplet interconversion in the radical pair (Woodward 2002). One would expect such a zero crossing in the response when the difference in electron Zeeman interactions of the two radicals in the applied magnetic field, Δ*ν*, is comparable to the hyperfine interactions. Using the *g* values of flavin (*g*≈2.0032; Ehrenberg *et al*. 1967; Műller *et al*. 1970) and tryptophan (*g*≈2.0027; Un 2005) radicals (which constitute the putative radical pair in cryptochrome), Δ*ν* in a 100 mT applied field is approximately 700 kHz which is small compared with the effective hyperfine interaction in this radical pair (approx. 100 MHz). In weaker applied magnetic fields Δ*ν* is proportionately smaller. It therefore seems inconceivable that the Δ*g* mechanism could compete with the dominant hyperfine mechanism of singlet–triplet interconversion: a field in excess of 1 T would be required for this to occur.

A further possible difference between the various laboratories could be the amplitude of the 50/60 Hz background fields experienced by the seedlings. We measured an upper limit of 5 nT inside the Microclima plant growth chamber; it is difficult to imagine how such a weak background could abolish the effect of a static field, which is five orders of magnitude stronger (500 μT).

As described in §2, there were other subtle disparities between our experimental procedures and those of Ahmad *et al*. (2007; and possibly also other unidentified differences), which might just explain the discrepancy between the two sets of results. We think this unlikely, but if it were the case it would nevertheless call into question the generality of the earlier results if they require conditions that are so specific and hard to repeat.

It is difficult to discern a systematic difference in the hypocotyl lengths of the seedlings grown in Oxford, Paris and Frankfurt that might shed light on our failure to replicate the earlier results. For the plants grown in the dark in an approximately 50 μT magnetic field, our mean hypocotyl lengths were 7.2, 9.0 and 10.2 mm for the three batches of seeds, and an overall mean of 9.0 mm for the 11 plates taken together (table 2). These values are comparable with the means (over three plates) found in Paris (9.7 mm) and Frankfurt (8.0 mm; Ahmad *et al*. 2007). Four out of five experiments using the batch of seeds labelled *C* (table 1) had longer hypocotyls in the dark at 50 μT than the six labelled *A* or *B*, and four of the five had shorter hypocotyls under blue light at 500 μT than at 50 μT. Although seedlings that germinate earlier and so have more time to grow might be expected to have larger magnetic responses, the effects for batch *C* (values of *Δ*; table 2) are considerably smaller than those found consistently in Paris and Frankfurt and were not statistically significant. Had the hypocotyls of the plants grown in Oxford been on average approximately 30 per cent shorter (as they were in Paris) at 500 than at 50 μT, the differences in all cases would almost certainly have been judged significant. Finally, the mean reductions in hypocotyl length produced by 20 W m^−2^ blue light at approximately 50 μT (46, 52 and 66% for the three seed batches) were also comparable with the Paris (65%) and Frankfurt (49%) figures. There are thus no clear differences in hypocotyl lengths at approximately 50 μT in the three laboratories. This appears to rule out the possibility, mentioned above, that differences in temperature between the exposed and control seedlings could have been responsible for an apparently positive magnetic field effect.

Similar experiments have now been conducted in three laboratories and reported in two papers: Ahmad *et al*. (2007) and here. Taking all the results at face value after appropriate analysis (or reanalysis), it is clear that plant growth responses to the imposed magnetic field treatments are highly variable. This is perhaps to be expected for biological material in which developmental processes *in planta* have a stochastic component. In cases where much the same experiments have been performed on much the same biological material more than once, it would seem most appropriate to draw conclusions on the basis of the combined results. Viewed in this way, it is difficult to avoid the conclusion that any case for rejecting the null hypothesis that our experiments were designed to test has been weakened.

Finally, we comment on the significance of our negative results for the continued study of the magnetic sensitivity of cryptochromes and the chemical compass mechanism of avian magnetoreception (Ritz *et al*. 2000). It is evident from our unpublished work that the magnetic responses of various members of the photolyase/cryptochrome family *in vitro* are strongly dependent on experimental conditions, including solvent viscosity. Little is known about the microscopic environment of the cryptochromes involved in regulating hypocotyl growth, gene expression and anthocyanin accumulation in plants. Moreover, one can therefore only speculate about the environment of an avian cryptochrome in a magnetoreceptor cell, although it seems clear in the latter case that the protein molecules must be both immobilized and aligned in order to show the anisotropic magnetic field effects essential for a compass detection mechanism (Ritz *et al*. 2000). There is no reason to think that the cryptochromes involved in blue-light growth inhibition in plant cells would need to have their motions similarly restricted. There are also no grounds for expecting the conditions that give strong magnetic field responses from isolated purified proteins *in vitro* to be necessarily similar to those in a plant or bird cell. The absence of detectable magnetic field responses on cryptochrome-mediated plant growth is therefore not incompatible with the existence of significant magnetic responses from cryptochromes *in vitro* or the sensitive detection of the geomagnetic field by cryptochromes in a bird's retina.
